# Exosomal miRNA-128-3p from mesenchymal stem cells of aged rats regulates osteogenesis and bone fracture healing by targeting *Smad5*

**DOI:** 10.1186/s12951-020-00601-w

**Published:** 2020-03-16

**Authors:** Tao Xu, Yongjun Luo, Jiaxing Wang, Ning Zhang, Changjiang Gu, Linwei Li, Dingfei Qian, Weihua Cai, Jin Fan, Guoyong Yin

**Affiliations:** Department of Orthopaedics, The First Affiliated Hospital of Nanjing Medical University, Nanjing, 210029 Jiangsu China

**Keywords:** Exosomes, miR-128-3P, Smad5, Mesenchymal stem cells, Bone fracture

## Abstract

Transplantation of mesenchymal stem cells (MSCs) has been considered an effective therapeutic treatment for a variety of diseases including bone fracture. However, there are associated complications along with MSCs transplantation. There is evidence to show that exosomes (Exos) derived from MSCs exert a similar paracrine function. In addition, repair capabilities of MSCs decline with age. Hence, this study aims to confirm whether the Exos protective function on osteogenic differentiation and fracture healing from aged MSCs was attenuated. This information was used in order to investigate the underlying mechanism. MSCs were successfully isolated and identified from young and aged rats, and Exos were then obtained. Aged-Exos exhibited significantly attenuated effects on MSCs osteogenic differentiation in vitro and facture healing in vivo. Using miRNA array analysis, it was shown that miR-128-3p was markedly upregulated in Aged-Exos. In vitro experiments confirmed that Smad5 is a direct downstream target of miR-128-3p, and was inhibited by overexpressed miR-128-3p. A series gain- and loss- function experiment indicated that miR-128-3P serves a suppressor role in the process of fracture healing. Furthermore, effects caused by miR-128-3P mimic/inhibitor were reversed by the application of Smad5/siSmad5. Taken together, these results suggest that the therapeutic effects of MSCs-derived Exos may vary according to differential expression of miRNAs. Exosomal miR-128-3P antagomir may act as a promising therapeutic strategy for bone fracture healing, especially for the elderly.

## Introduction

Fractures are one of the most common injuries of the musculoskeletal system, [1, 2]. However, approximately 5–10% of patients suffered from delayed or non-healing resulting from the impaired fracture repair [3, 4]. Prolonged healing and ongoing treatment results in an undue economic burden on patients as well as poor life quality. Fracture healing is a complicated process, which is influenced by many factors [5]. Several cell types are involved in the dynamic modeling and remodeling process of bone tissue [6]. Among all the potential factors involved in bone metabolism, the differentiation capacity of bone marrow-derived mesenchymal stem cells (MSCs) is recognized to play quite a critical role [7–9]. Lineage specific differentiation of bone marrow stem cells is crucial to tissue repair. Bone tissue regeneration requires coordinated enrollment of osteoblasts and osteoclasts that are terminally differentiated from MSCs. In particular, the osteoblast, of mesenchymal origin, is pivotal during bone remodeling and skeletal development [10]. Transplantation of MSCs into target tissues has been proven effective in many disease models, including the promotion of osteogenesis in fractures [11, 12]. However, direct transplanting of MSCs has non-negligible limitations, such as low survival rate, immune rejection and inducing tumor formation.

Exosomes (Exos) are 50–100 nm diameter extracellular vesicles secreted by many cell types. Exos originate from invagination of endosomal membranes and are released into the extracellular space [13]. Exosomes participate in intercellular communication by transport of functional biomolecules, such as proteins, cytokines, mRNAs, and micro-RNAs, which differ depending on the producer cells. Specific surface ligands make it possible for Exos to combine with target cells, deliver contents, and eventually produce specific biological effects [14]. Exos may be considered as an important paracrine secretion of cells [15]. Furuta T et al. [16] has demonstrated that MSCs-derived exosomes could promote fracture healing in mice, which suggests that Exos transplantation may serve as a prospective substitutional strategy for MSC-related treatment in conditions of bone non-union or delayed fracture healing.

Micro-RNAs (miRNAs) are a class of highly conserved small noncoding RNAs that regulate gene expression post-transcriptionally. Micro-RNAs modulate a series of biologic processes involved in tumor progression, metabolic diseases, stem cell self-renewal, development, differentiation, and growth [15, 17–19]. Increasing studies have found that miRNAs may function in the induction of osteoblast differentiation and bone formation. For example, Li et al. [20] showed that miRNA-21-5p promoted osteoblast differentiation in a hypoxic environment. Likewise, Xue et al. [21] suggested that miR-125b could suppress osteoblastic differentiation through an NKIRAS2 and NF-κB pathway. Taken together, exploring the underlying mechanism of osteoblast differentiation induced by Exos-delivered miRNA may help us to better understand bone loss diseases and thereby develop new treatment strategies.

Aging has been correlated with bone loss and delayed fracture healing [22]. Age-related osteoporosis is associated with significantly reduced bone formation resulting from decreased numbers and degraded osteogenic ability of MSCs [23, 24]. Nevertheless, the specific age-related mechanism of these observations remains unclear. Using miRNA array analysis, we found that miR-128-3p was one of the top miRNAs in Aged-Exos. Interestingly, the expression levels of miR-128-3p were significantly higher than in Young-Exos. We predicted the downstream target genes of miR-128-3p using Targetscan database and found several potential targets which might affect fracture healing. Among these candidate genes, Smad5 (Smad family member 5), as a downstream signal molecule of bone morphogenetic proteins (BMPs)*,* was of particular interest. BMPs are osteogenic genes that have been shown to exhibit the strongest osteogenic activity in vivo and in vitro [25, 26]. Expression of Smad5 parallels changes in expression of endogenous BMP activity [27]. We found in this study that the protective effects of exosomes derived from aged rats’ MSCs on fracture healing in vivo and osteogenic differentiation in vitro were attenuated by upregulated miR-128-3p. These findings may indicate a novel mechanism of MSCs-derived exosomes that affects bone fracture healing and provide a promising avenue for fracture treatment, especially for the elderly.

## Materials and methods

### Animals

All rats in this study were acquired from the Laboratory Animal Research Center of Nanjing Medical University. Three-month-old male Sprague–Dawley (SD) rats were used for the femoral fracture model. Four-week-old and 72-week-old SD rats were used for MSCs isolation, representing young and aged rats, respectively. This study was approved by Animal Experimentation Ethics Committee of Nanjing Medical University and all the procedures were conducted abiding the Guidelines for the Care and Use of Laboratory Animals.

### Cell culture

Rat MSCs were obtained from young (4-week-old) and old (72-week-old) rats’ bone marrow and seeded in 100 mm-diameter cell culture dishes with 10 ml specific culture medium at 37 ℃ with 5% CO_2_. To ensure exosomes-free culture media, exosome-depleted fetal bovine serum (FBS)-containing medium was used (EXO-FBS-250A-1; System Biosciences, Mountain View, CA, USA). The MSCs were washed with PBS after 48 h and passaged at 80% confluency and all experiments were conducted using the 3rd-5th passage.

### Identification of MSCs

The morphology of aged and young MSCs was observed via microscopy (Axio Observer 3.1, Zeiss, Oberkochen, Germany) and the immunophenotypes of MSCs (CD90/CD44/CD45-/CD34-) were characterized by LSR II flow cytometry (BD Bioscience). The data were analyzed by Flowjo X 10.0 (Tree Star Inc.). All experiments were conducted in triplicate. MSCs osteogenic differentiation were measured via Alkaline Phosphatase (ALP) staining and Alizarin Red staining. Toluidine Blue staining and Oil Red O staining were conducted for the identification of chondrogenic and adipogenic differentiation. For identification of MSCs differentiation, cells were induced for at least 14 days with different treatment. Then cells were fixed in 0.4% formaldehyde (Klinipath, Olen, Belgium) for 5 min, washed with PBS and deionized water, and stained with 2% Alizarin Red solution, 0.5% Toluidine Blue solution or saturated Oil Red O solution for 10 min, then rinsed with distilled water. ALP activity was evaluated using an ALP staining kit (Beyotime Institute of Biotechnology, China). The osteogenic differentiation and mineralization level of MSCs was evaluated according to the intensity of ALP and Alizarin Red staining.

### Exosome isolation and purification

MSCs were incubated for 48 h after reaching 80% confluency, replacing the media with exosome-depleted FBS. Then, the supernatant was collected and centrifuged successively at 300 × *g* for 10 min and 2,000 × *g* for 10 min at 4 °C. After centrifugation, the cell supernatant was filtered through a 0.22-μm sterile filter (Steritop™ Millipore, Burlington, MA) and applied to the upper compartment of an Amicon Ultra-15 Centrifuge Filter Unit (Millipore). Then the supernatant was centrifuged at 4,000 × *g* till the volume decreased to 200 μl in the upper compartment. The ultra-filtered supernatant was then washed twice with PBS and re-filtered to another 200 μl. The liquid was loaded onto the top of a 30% sucrose/D2O cushion in a sterile Ultra-Clear™ tube (Beckman Coulter, Asphalt, CA, USA), followed by centrifugation at 100,000 × *g* for 60 min at 4 °C with an optima L-100 XP Ultracentrifuge (Beckman Coulter). Exosomes were stored at -80 °C.

### Exosome identification

The morphology of the Exos was observed under a transmission electron microscope (TEM; Tecnai 12, Philips, The Netherlands). The diameter distributions of both Young-Exos and MSC-Exos were analyzed via Nanosight LM10 System (Nanosight Ltd, Navato, CA). Specific exosome surface markers like CD81 and CD63 were examined using LSR II flow cytometer (BD Bioscience) and the data were also analyzed by Flowjo X 10.0 (Tree Star Inc.).

### Osteogenic differentiation of MSCs

Osteogenic differentiation of MSCs isolated from 4-week-old rats was performed 24 h after the incubation of Exos or transfection of miRNA mimics or inhibitors. Briefly, the original medium was replaced by osteogenesis-induction medium (20 mM β-glycerophosphate, 50 mM L-ascorbic acid-2-phosphate, and 1 nM dexamethasone in complete medium). The induction medium was refreshed every 72 h together with 200ul Young-Exos (200 µg/mL), Aged-Exos (200 µg/mL) or PBS. The mimics or inhibitor transfections were performed after the induction medium was refreshed. Total RNA was extracted at 7 and 14 days after induction for qRT-PCR analysis.

### Fracture model experiments

Rat femoral fracture model was conducted as follows. Briefly, rats were generally anesthetized before we made a 2-cm incision. A 1-mm-diameter Kirschner’s wire was inserted into the femur through the patellar tendon, then a middle femoral fracture was conducted using a bone forceps. After surgery, the rats were randomly assigned to 3 groups: PBS, Young-Exos or Aged-Exos (n = 12/group). PBS (200 μL), Young-Exos or Aged-Exos (200 μg exosomes total protein settled in 200 μL of PBS) were respectively injected around the fracture area. The entire fracture model was finished after the closure and suture of the incision. For the first 3 days post-operatively, pain levels of the rats were controlled by injecting buprenorphine every day. All efforts were made to help rats suffer the least and all surgical procedures above-mentioned were performed in a sterile environment.

### Microcomputed tomography (micro-CT) imaging

Four rats from each group were randomly sacrificed at 2, 3 and 4 weeks after the surgery respectively. Femurs were removed and fixed in 4% paraformaldehyde for 24 h. Fracture callus was evaluated using a micro-CT system (SkyScan 1172, Bruker, Belgium) at a resolution of 9 µm with 50 kV and 200 µA. We constructed 3-dimensional structures and analyzed the mineralized callus volume (CV) as bone morphometric parameter. The ratio of bone volume (BV) and total volume (TV) was also calculated after 4 weeks. Then the femurs were decalcified in 10% ethylenediaminetetraacetic acid (EDTA) for 3–4 weeks, and embedded in paraffin.

### Western blotting analysis

The cultured MSCs were lysed with RIPA lysis and extraction buffer (KeyGEN Biotechnology, China) to acquire bone-related proteins such as Collagen I (Col I), Alkaline phosphatase (ALP) and Runt-related transcription factor 2 (Runx2). The concentration of these proteins was determined via the bicinchoninic acid (BCA) protein quantification kit (Pierce, Waltham, MA, USA). Equal amounts of proteins were separated on an 10% SDS-PAGE gel by electrophoresis, and then transferred to poly-vinylidene fluoride (PVDF) membranes (EMD Millipore Corp., Burlington, MA). The membranes were blocked with bovine serum albumin (5%, v/v) for 2 h at room temperature prior to incubating overnight at 4 °C in respective primary Abs. After washing with Tris-Buffered Saline Tween-20 (TBST), membranes were incubated in appropriate secondary Abs for 2 h at room temperature. The membrane was washed with TBST after incubation, then washed and reacted with a chemi-luminescent substrate according to the manufacturer’s instructions (Thermo Scientific, Waltham, MA, USA). The reacting bands were visualized using ECL reagent (Thermo Fisher Scientific) and imaged using ImageJ (National Institutes of Health, Bethesda, MD, USA).

The glyceraldehyde-3-phosphate dehydrogenase (GAPDH) was used as an internal control. The primary antibodies used in the experiments were anti-ALP (1:1000; Abcam, USA), anti-Runx2 (1:1000; Abcam, USA), anti-Col I (1:1000; Abcam, USA), anti-*Smad5* (1:1000; Abcam, USA) and anti-GAPDH (1:1000; Abcam, USA).

### Quantitative real-time PCR (qRT-PCR)

Total RNA was isolated from exosomes or cells induced for 7 and 14 days using Trizol reagent (Invitrogen, Carlsbad, CA, USA). Callus tissues collected from fracture sites at 14, 21 and 28 days post-operation were also used to extract RNA with the same Trizol reagent. A Reverse Transcription System (Toyobo, Osaka, Japan) was used to reverse-transcribe messenger RNA to complementary DNA (cDNA) and qRT-PCR was performed with SYBR Green PCR master mix (Applied Biosystems, Foster City, CA) on an ABI 7900 fast real-time PCR system (Applied Biosystems, Carlsbad, USA). GAPDH or U6 were used as an internal control to normalize the expression levels of mRNA or miRNA. The relative expression levels were then calculated using the 2^−ΔΔCT^ method. Bulge-loop™ miRNA qRT-PCR Primer Sets (one RT primer and a pair of qPCR primers for each set) specific for miR-128-3p is designed by RiboBio Co, Ltd. (Guangzhou, China). The primers for U6, GAPDH, ALP, Col I and Runx2 were purchased from RiboBio (Guangzhou, China) and the specific primer sequences were showed in Table 1.

**Table 1:** The primers used for PCR

Gene name	Primer (5′ to 3′)	Primer sequence
U6	Forward	CTCGCTTCGGCAGCACA
	Reverse	AACGCTTCACGAATTTGCGT
Runx2	Forward	CGAATAACAGCACGCTATTAA
	Reverse	GTCGCCAAACAGATTCATCCA
ALP	Forward	TAGTGAAGAGACCCAGGCGCT
	Reverse	ATAGGCCTCCTGAAAGCCGA
Col I	Forward	CCAGAA GAACTGGTACATCAGCAA
	Reverse	CGCCATACT CGAACTGGAATC
GAPDH	Forward	TCATGGGTGTGAACCATGAGAA
	Reverse	GGCATGGACTGTGGTCATGAG
Smad5	Forward	CGGTAGCCAACTGACTTTGAGT
	Reverse	ACCTTGTTTCCAGCCCAACA

### miRNA expression and transfection

miR-128-3p-mimics, miR-128-3p-mimics-NC (NC-mimics), miR-128-3p-inhibitor and miR-128-3p-inhibitor-NC (NC-inhibitor) were constructed by RiboBio (Guangzhou, China) for the MSCs osteogenic differentiation assay in vitro. The MSCs were transfected with mimics or inhibitors at a density of 4 × 10^5^ each well in six-well plates using Lipofectamine 3000 reagent (Invitrogen). miR-128-3p-antagomir and miR-128-3p-antagomir-NC (NC-antagomir) were constructed by RiboBio (Guangzhou, China) to treat young or aged rats’ femoral fracture. We injected 20 nmol antagomir around the fracture for each rat on day 1 post-operation and then every 3 days until euthanasia.

### miRNA array analysis

To quantify miRNA in Exos derived from MSCs of young and aged rats (Young-Exos and Aged-Exos), microarray analysis was conducted with mirVana miRNA Bioarray V9.2 (Ambion). miRNA was purified from 22 μg total RNA obtained from Young-Exos or Aged-Exos using the flash PAGE system (Ambion) in triplicate. Samples were analyzed according to methods previously described [28]. With the Microarray Data Analysis Tool (Filgen, Inc.), the miRNA array data were normalized using global normalization. The selection criteria of miRNA referred to the results based on miRNA array PCR data.

### Luciferase reporter assay

Wild-type Smad5 luciferase reporter vectors (Wt-Smad5) were constructed by cloning the fragment of Smad5 into the downstream region of the renilla psiCHECK2 vector (Promega, WI, USA). The seed region of Smad5 was mutated (Mut- Smad5) to remove the complementarity to miR-128-3p in order to generate the Smad5 mutant reporter. Wt-Smad5 or Mut- Smad5 was transfected into HEK293T cells (ATCC, USA) together with miR-128-3p mimics or miR-128-3p inhibitor. Luciferase activity was measured 48 h post-transfection with the Dual Luciferase Reporter Assay System (Promega) and normalized to the firefly luciferase activity.

### Immunohistochemistry

Femurs were fixed in 4% paraformaldehyde and embedded with paraffin. After endogenous peroxides and proteins were blocked, 6-μm-thick sections were incubated with primary anti-*Smad5* antibody (Abcam, Cambridge, UK) overnight at 4 °C. Sections were incubated with secondary antibody at 37 °C for 1 h and with 3,3-diaminobenzidine (DAB) for 3 min.

### Statistical analysis

All independent experiments were performed in triplicate and all quantitative data were expressed as mean ± SD. Statistical analysis was performed via GraphPad software 7.0 and SPSS 19.0. Two-tailed Student's t-test was used for comparisons between two groups and One-Way Analysis of Variance (ANOVA) with Bonferroni post hoc test were used to calculate the P-values for multiple comparisons. A value of P < 0.05 was considered as statistically significant and represented as the symbols “*”.

## Results

### Identification of MSCs

**Fig. 1:**
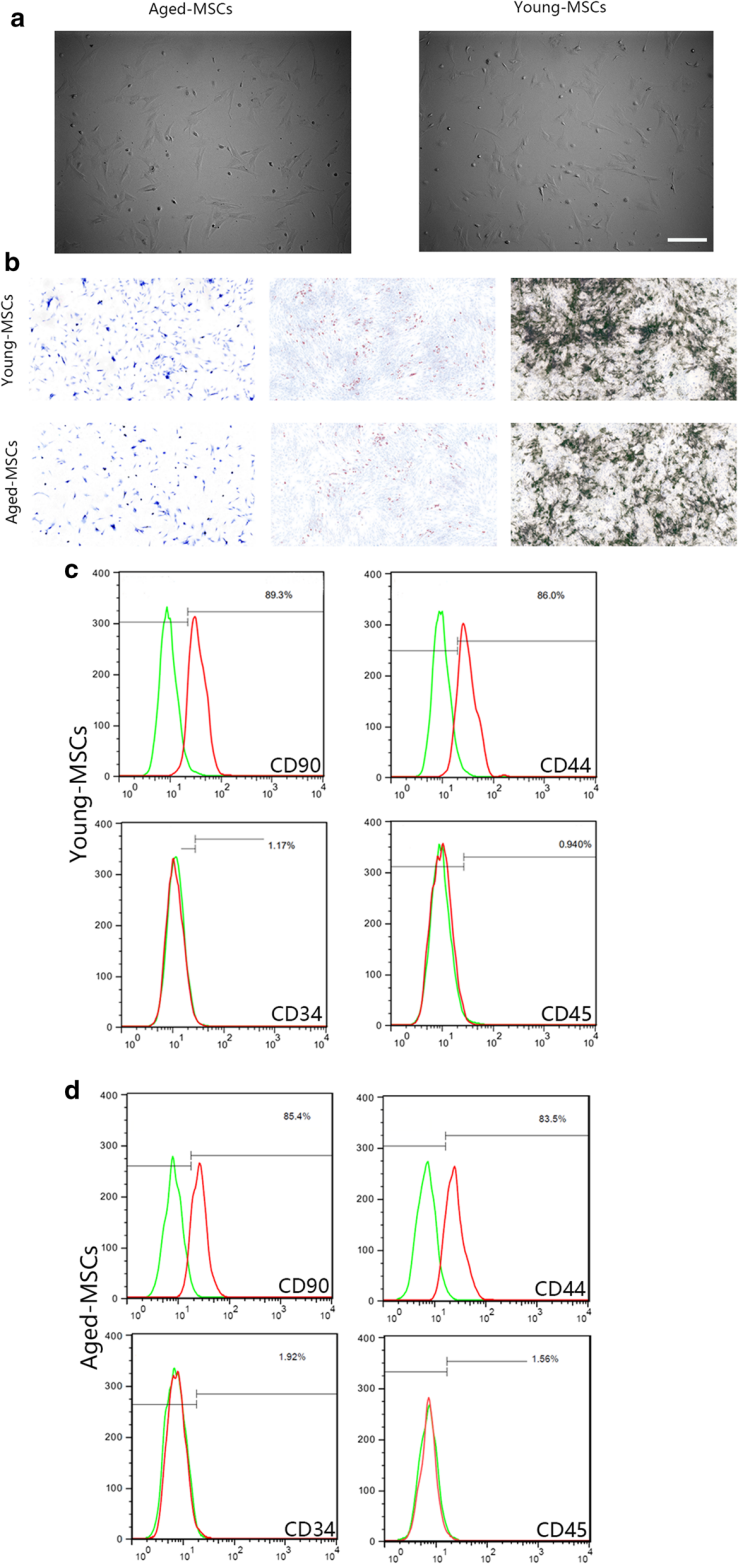
**a** MSCs adopted a spindle-like morphology, whereas aged MSCs demonstrated a flattened morphology compared to young MSCs (scale: 100 μm). **b** Toluidine Blue, Oli Red O and ALP staining of MSCs (scale: 100 μm). **c**, **d** MSCs were positive for CD44 and CD90 but negative for CD34 or CD45

MSCs were obtained from femurs of 4-week-old and 72-week-old rats as described above. MSCs were identified by morphology and flow cytometry at passage number three. Cells exhibited a spindle-like shape (Fig. 1a) but aged MSCs had a flattened shape compared to young MSCs. The chondrogenic, adipogenic and osteogenic differentiation potential of MSCs were verified with Toluidine Blue, Oli Red O, and ALP staining, respectively (Fig. 1b). Flow cytometry analysis confirmed that MSCs were positive for CD44 and CD90, but negative for CD34 or CD45 (Fig. 1c, d).

### Characterization of exosomes

**Fig. 2:**
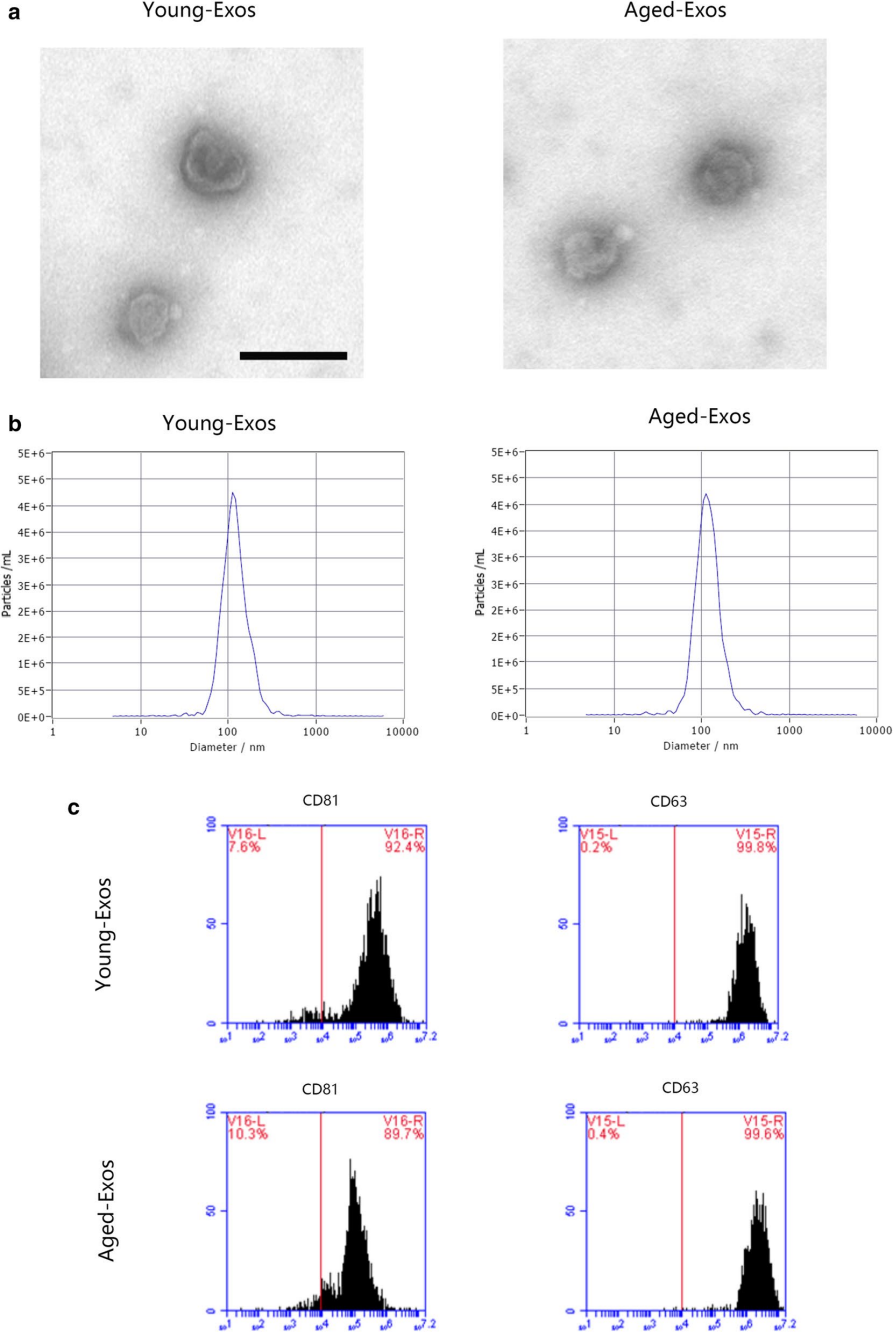
**a** The morphology of Young-Exos and Aged-Exos (scale: 100 nm). **b** The diameter of Young- and Aged-Exos, ranging from 50 to 150 nm. **c** Specific surface markers of exosomes, including CD63 and CD81

The Exos were absorbed by MSCs as showed in Additional file 1: Figure S1. Other characterization includes morphological observation by TEM, dynamic light scattering (DLS) analysis, and Flow Cytometry analysis. As Fig. 2a shows, the morphology of Young-Exos showed a rounded shape under TEM, which resembled the morphology of Aged-Exos. The diameter size of these particles was evaluated by DLS analysis, both ranging from 50 to 150 nm (Fig. 2b). Specific surface markers of exosomes, including CD63 and CD81 was detected to be positive in Exos by Flow Cytometry analysis (Fig. 2c). These findings were similar to previous studies [29–31] and indicated a successful isolation and identification of the exosomes derived from young and aged MSCs.

### Young-Exos promote MSCs osteogenesis differentiation in vitro

**Fig. 3:**
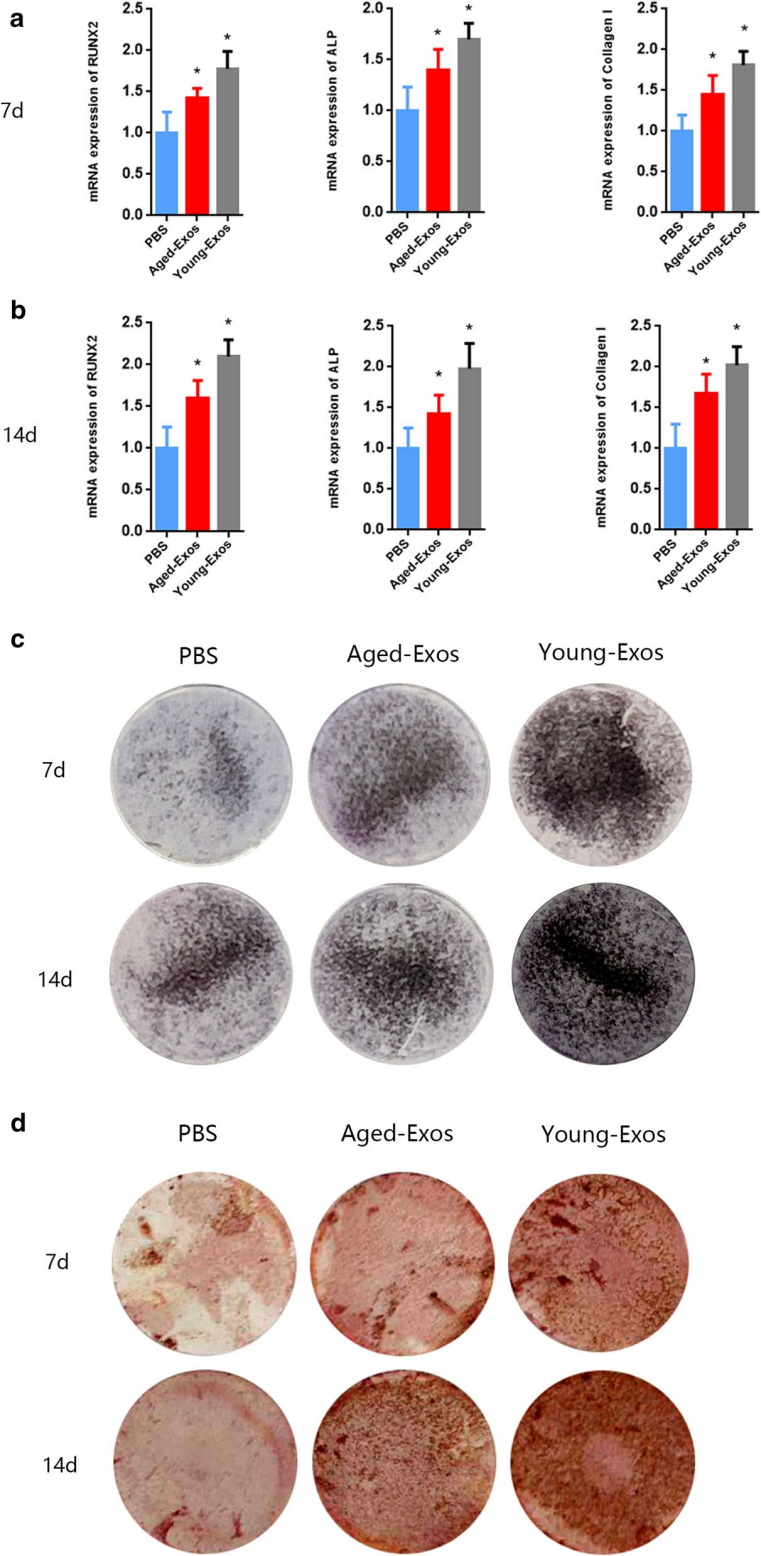
**a**, **b** The mRNA expression levels of Runx2, ALP and Col I (n = 5, PBS vs Age-Exos, P < 0.05, Age-Exos vs Young-Exos, P < 0.05). **c**, **d** ALP staining and Alizarin Red staining after 7 or 14 days of incubation with Exos

The next step was to investigate whether Exos induced from young and aged MSCs have therapeutic effects on cell osteogenesis differentiation in vitro. MSCs of 4-week-old rats were cultured using osteogenic differentiation induction medium together with 200 µg/mL Aged-Exos, Young-Exos, or equal quantities of PBS, respectively. The expression of osteogenesis-related genes (Runx2, ALP and Col I) were examined every seven days using qRT-PCR. As shown in Fig. 3a, b, the highest mRNA expression levels of Runx2, ALP and Col I was found in the Young-Exos group, suggesting a remarkable increase in osteogenic ability of MSCs incubated with Young-Exos. Both the ALP and Alizarin Red staining showed the strongest intensity in MSCs after 7 or 14 days of incubation with Young-Exos (Fig. 3c, d).

### Exosome uptake experiment

Dil solution (Molecular Probes, Eugene, OR, USA) was added to the solution contained Young-Exos or Aged-Exos (1: 200) and incubated at 4 °C for 15 min. Then adding PBS and ultra-centrifugate as mentioned above to remove excess dye. Repeat this step three times. Fluorescently labeled Young-Exos and Aged-Exos were obtained and then respectively co-cultured with MSCs for 24 h, fixed with 4% paraformaldehyde for 15 min, rinsed 3 times by PBS. Finally, the uptake of exosomes was observed with laser confocal microscopy.

### Bone fracture healing in rats after Exos application

**Fig. 4:**
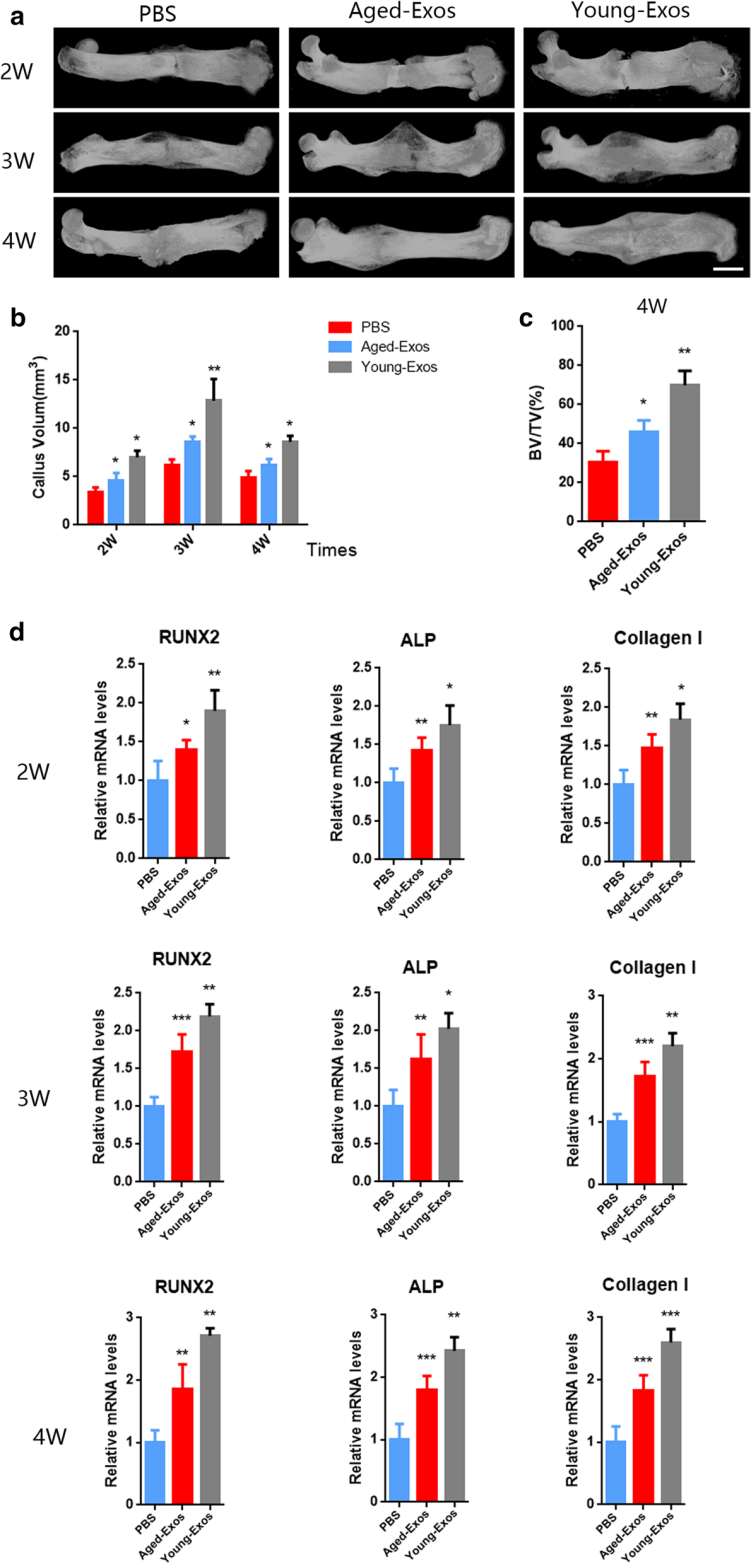
**a**, **b** Micro-CT images and Callus volumes (scale: 1 mm). **c** BV/TV on day 28. **d** The mRNA levels of Runx2, ALP and Collagen I in calluses (n = 4, PBS vs Age-Exos, P < 0.05, Age-Exos vs Young-Exos, P < 0.05)

As demonstrated above, Young-Exos were found to accelerate MSCs osteogenesis differentiation in vitro. Our micro-CT examination analyzed different callus formations after application of PBS, Aged-Exos, or Young-Exos. In Fig. 4a, apparent fracture gaps were observed 2 weeks postoperatively in all the groups but callus volumes were found larger in the Young-Exos group compared to the other two groups. Similar results were detected 3 and 4 weeks postoperative. In the Young-Exos group, fracture gaps were covered by a callus, and volumes of newly formed calluses were larger as well. According to the results from Micro-CT examination, transplantation of Young-Exos increased the CV significantly (Fig. 4b). Micro-CT reconstruction on post-operative day 28 showed that BV/TV was highly elevated in rats treated with Young-Exos (Fig. 4c). As showed in Fig. 4d, the expression level of Runx2, ALP and Col I in calluses were upregulated as well after administration of Young-Exos after 2, 3 and 4 weeks.

### The miR-128-3p is upregulated in Aged-Exos

**Fig. 5:**
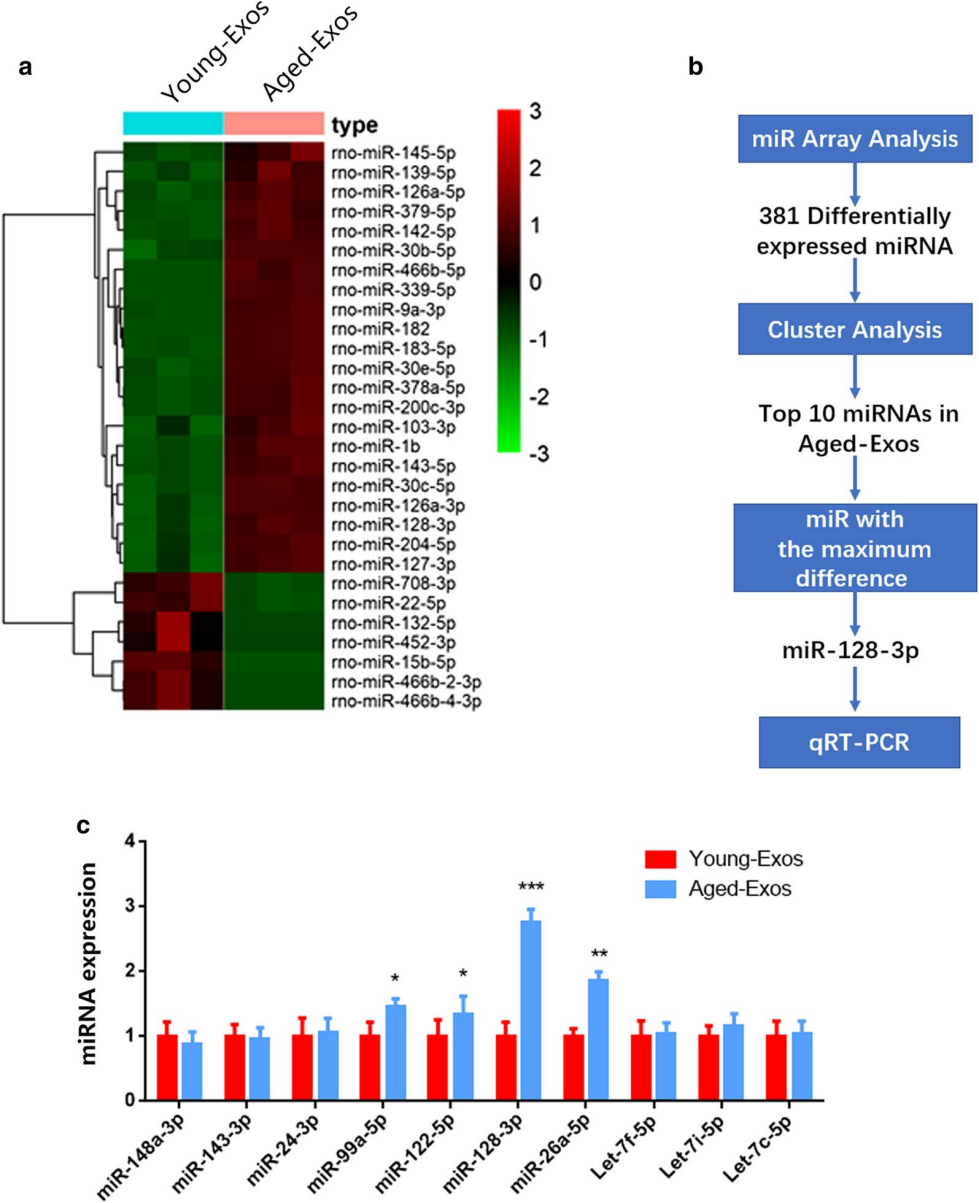
**a** The heat map showed that miR-128-3p was upregulated in Aged-Exos. **b** The schematic diagram of microRNA screening and selection. **c** qRT-PCR showed that miR-128-3p was the most upregulated miRNAs in the Aged-Exos among the top 10 miRNAs

It was shown that Young-Exos enhanced osteogenesis in vitro and promoted fracture healing in vivo when compared to Aged-Exos and PBS controls. It has been concluded that exosomal miRNAs regulate multiple biological functions functioning in intercellular communication [32–34]. Therefore, Exos were collected from the supernatant of aged and young MSCs and the miRNA levels were evaluated by miRNA array analysis. Heat map analysis (Fig. 5a) showed that miR-128-3p was upregulated in Aged-Exos. The top ten miRNAs in Aged-Exos were selected and qRT-PCR was conducted to confirm the miRNA levels. Figure 5c showed that miR-128-3p was the most upregulated miRNAs in the Aged-Exos compared to the Young-Exos.

### Exosomal miR-128-3p regulates Smad5 by directly targeting the 3′-UTR.

**Fig. 6:**
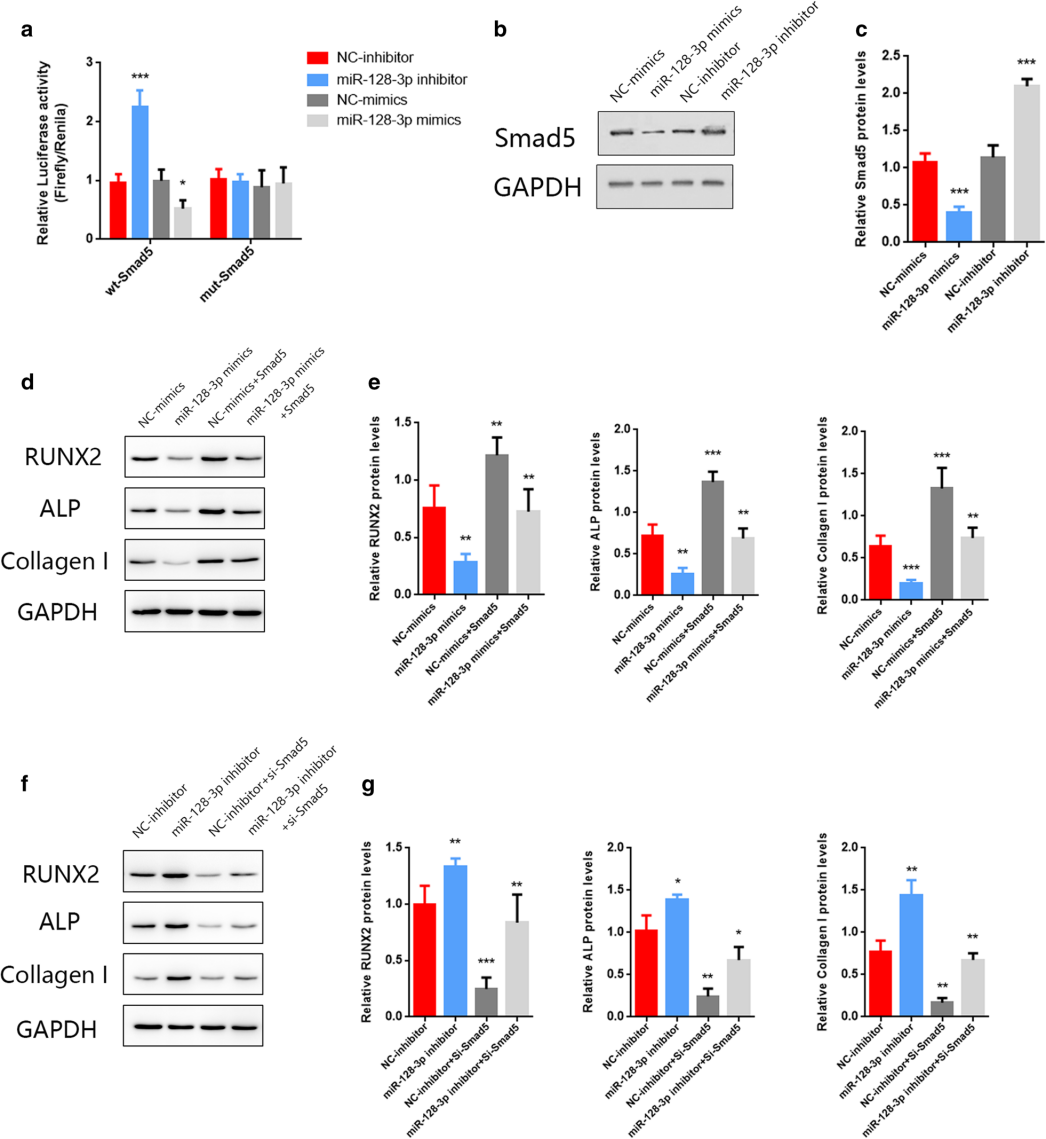
**a**. The relative luciferase activity was lessened when miR-128-3p mimics were co-transfected with Wt-Smad5 luciferase construct (n = 6, NC-mimics vs miR-128-3p mimics, P < 0.05), but not with the Mut-Smad5. An enhanced luciferase activity was observed when the miR-128-3p inhibitor was co-transfected with Wt-Smad5 (NC-inhibitor vs the miR-128-3p inhibitor, P < 0.05). **b**, **c** After 14 days of administration of the miR-128-3P inhibitor, the protein levels of Smad5 in MSCs were markedly increased (n = 4, P < 0.05). In contrast, decreased Smad5 expression was found in MSCs incubated with the miR-128-3P mimics (n = 4, P < 0.05). **d**, **e** Western blot analysis of Runx2, ALP and Col I protein product levels. Upregulating *Smad5* rescued the negative effects of MSCs osteogenic differentiation caused by the miR-128-3P mimics (n = 4, P < 0.05). **f**, **g** Osteogenic differentiation was accelerated when aged MSCs were incubated with the miR-128-3P inhibitor and these positive effects could also be reversed by silencing Smad5 (n = 4, P < 0.05)

Since the miR-128-3p expression was confirmed as abundant in Aged-Exos, the next step was to predict the potential target genes of miR‐128‐3p. According to predictions from the online database Targetscan, Smad5 stood out among all other candidate target genes. Smad5 is one of the downstream genes of BMPs and has paralleled expression levels with endogenous BMP activity. The BMP/Smad axis functions in cell osteogenesis and osseous development of multiple cell and tissue types. Next, the relationship between exosomal miR-128-3P and Smad5 was investigated using a series of in vitro experiments. The seed region of the Mut-Smad5 was reconstructed as shown in Additional file 2: Figure S2. The Wt-Smad5 or Mut-Smad5 were transfected into HEK293T cells together with miR-128-3p mimics or miR-128-3p inhibitor. The transfection efficiency of the mimics and inhibitor was verified by qRT-PCR (Additional file 3: Figure S3). Luciferase reporter assay was conducted to verify that the Smad5 3′UTR is a direct target for miR-128-3p. A wild-type Smad5 and mutant-type Smad5 luciferase reporter gene vector was constructed as shown in Additional file 2: Figure S2. The relative luciferase activity decreased when miR-128-3p mimics were co-transfected with Wt-Smad5, but not with the Mut-Smad5 (Fig. 6a). However, an enhanced luciferase activity was observed when the miR-128-3p inhibitor was co-transfected with Wt-Smad5. Using Western blotting analysis, elevated expressional levels of Smad5 were detected in MSCs after administration of the miR-128-3P inhibitors. As shown in Fig. 6b, c, after the administration of miR-128-3P inhibitor for 14 days, the protein levels of Smad5 in MSCs was markedly increased compared with levels in the NC-inhibitor group. In contrast, decreased Smad5 expression was found in MSCs incubated with the miR-128-3P mimics. In summary, we confirmed that miR-128-3P negatively modulates Smad5 by directly targeting its 3′-UTR.

### MiR-128-3P/Smad5 axis modulates osteogenic differentiation of MSCs in vitro

In the subsequent experiments in vitro, we investigated the effects of miR-128-3P and Smad5 on osteogenic differentiation ability of young and aged MSCs respectively. Firstly, we upregulated miR-128-3P level in young MSCs and a significant decrease of Runx2, ALP and Col I were detected by the Western blotting assays. Then upregulating Smad5 rescued the negative effects of MSCs osteogenic differentiation caused by the administration of miR-128-3P mimics (Fig. 6d, e). Similarly, we found that the osteogenic differentiation was accelerated when aged MSCs were incubated with miR-128-3P inhibitor and these positive effects could also be reversed by silencing Smad5 (Fig. 6f, g). For this part, we demonstrated that miR-128-3P exerted an inhibitory role on MSCs’ osteogenic differentiation and these effects could be abolished by Smad5.

### Alternation of miR-128-3P in vivo could affect the femoral fracture healing process in rats

**Fig. 7:**
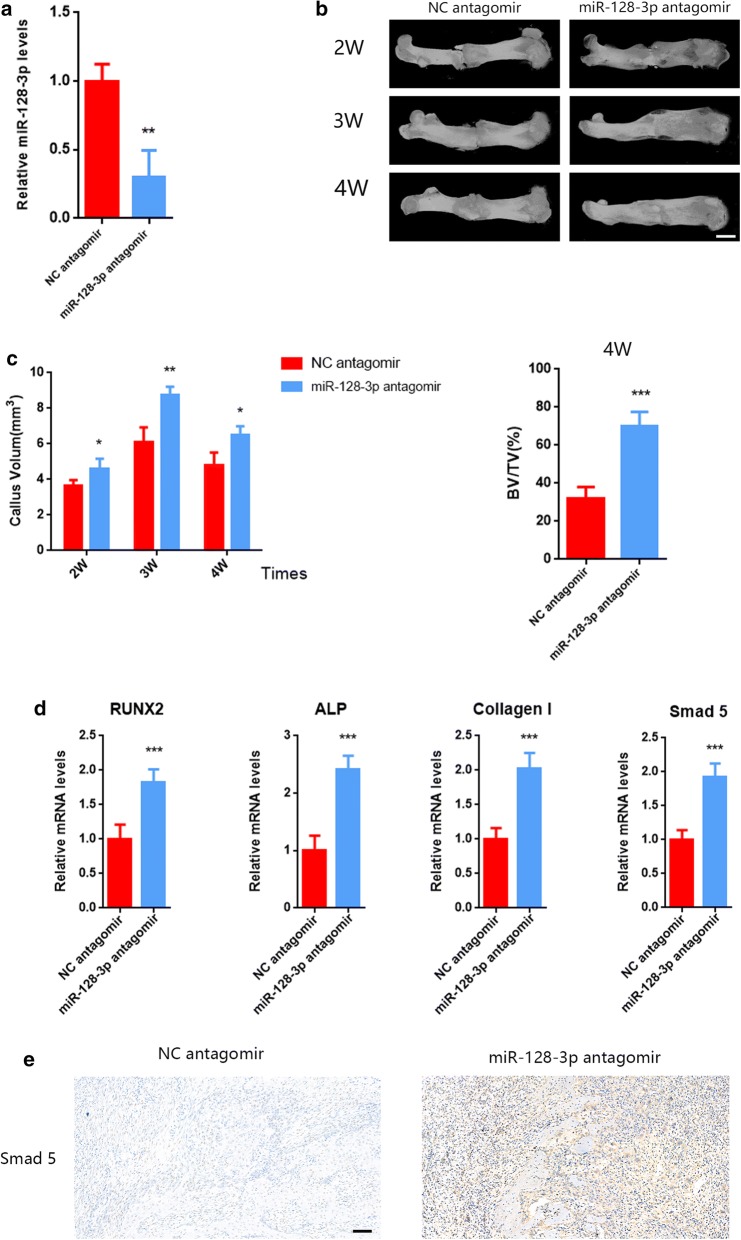
**a** The expression of miR-128-3P in fractured callus at day 14 (NC-antagomir vs miR-128-3P antagomir, P < 0.05). **b**, **c** The post-operative micro-CT reconstruction images at 2, 3 and 4 weeks. The CV and the BV/TV were increased by the miR-128-3P antagomir treatment (n = 4, NC-antagomir vs miR-128-3P antagomir, P < 0.05, scale: 1 mm). **d** The mRNA level of ALP, Runx2, Collagen I and Smad5 in the callus at day 28(NC-antagomir vs miR-128-3P antagomir, P < 0.05). **e** The results of immunohistochemistry (NC-antagomir vs miR-128-3P antagomir, P < 0.05, scale: 100 μm)

In order to further investigate the function of exosomal miR-128-3P on rats’ femoral fracture coalescence, both miR-128-3P antagomir and antagomir-NC were constructed by RiboBio (Guangzhou, China). Using the qRT-PCR assay, the expression of miR-128-3P in fractured calluses showed a suppression in the antagomir group at day 14 after local injection of antagomir around the fracture area (Fig. 7a). The post-operative micro-CT reconstruction images at 2, 3 and 4 weeks showed that, compared to the NC antagomir group, the fracture gaps were diminishing in the miR-128-3P antagomir group. Furthermore, the CV and the BV/TV showed a marked increased by miR-128-3P antagomir treatment as well (Fig. 7b, c). According to the qRT-PCR results, the mRNA levels of ALP, Runx2, Col I and Smad5 in the callus were upregulated by miR-128-3P antagomir at day 28 (Fig. 7d). Furthermore, the results of immunohistochemistry showed that the expression level of Smad5 was significantly upregulated in rats treated with miR-128-3P antagomir for 4 weeks (Fig. 7e). These data indicated an anti-osteogenic function of miR-128-3P in vivo via targeting Smad5.

## Discussion

With an aging society comes the prevalence of age-related diseases. alone such example is increased bone fractures among the elderly. Bone fracture healing ability differs greatly depending on the age of the organism. For example, numerous elderly people suffer from delayed bone fracture healing or non-union reaching up to 18.5% in some specific types of fractures [35]. The prolonged rehabilitation process after fracture may cause = severe complications and may even result in death [36–38]. Mesenchymal stem cells (MSCs) have great potential for self-replication and differentiation into several types of cells including osteoblasts [39]. Osteoblasts are of mesenchymal origin and function in skeletal development and bone formation [40, 41]. Additionally, MSCs can secrete various growth factors and angiogenic factors such as BMP-2 and vascular endothelial growth factor (VEGF) [42]. As a result, MSCs transplantation has been considered as a practical option for bone tissue engineering [43, 44]. However, the clinical application of MSCs is restricted by the invasive harvesting procedures, the dependence for carriers, as well as consideration of ethical and safety issues [45, 46]. More importantly, the biological effects of MSCs decline notably in age-related diseases. Given this situation, efficient applications of these stem cells for clinical disorders still have to cross may hurdles ahead (Additional file 4).

Exosomes (Exos) was first reported in 2010 as the active agent secreted by MSCs against myocardial ischemia reperfusion injury [47]. Afterwards, MSC-derived Exos were reported to exert therapeutic effects on a variety of diseases (osteonecrosis, ischemic diseases, spinal cord injury, and chronic cutaneous wounds) [48, 49]. Increasing evidence indicates that the reparative abilities of MSCs are derived from secretion and paracrine mechanisms [50–52]. Exos are known for their homogeneous therapeutic efficacy while overcoming the restrictions of direct cell transplantation. It has been demonstrated in several studies that Exos derived from MSCs could enhance the healing process in animal fractures or bone defect models [16, 53, 54]. The osteogenic differentiation ability of MSCs decays as the individuals get older. Biological information, such as proteins, mRNAs, and miRNAs contained in MSCs and MSC-derived Exos may differ due to distinct age status. In our preliminary in vivo studies, Exos derived from aged rats were observed with attenuated therapeutic function in a rat femoral fracture model. It was speculated whether Exos derived from young MSCs exerted more protective effects on the fracture healing in vivo and MSCs osteogenesis in vitro than those from aged MSCs. This was conducted in order to investigate the underlying mechanism.

Hence, in this study, several experiments were performed to verify our hypothesis. Firstly, we successfully obtained and identified MSCs from aged and young rats. It was demonstrated that both aged and young MSCs could differentiate into osteoblasts, chondrocytes, or adipocytes. The characteristics of aged MSCs resembled young MSCs with the exception of a more flattened morphology. Then, we isolated Exos from the supernatant of either aged or young MSCs. The TEM and NTA results showed high morphological similarities between the Aged-Exos and Young-Exos according to their shape, size, and density. Nevertheless, local injection treatment revealed that Young-Exos promoted fracture healing more abundantly than Age-Exos. Furthermore, in vitro studies showed that the osteogenesis of MSCs were significantly enhanced after incubation with Young-Exos. A recent study conducted by Taisuke et al. [16] demonstrated that MSCs-derived Exos promoted fracture healing in a mouse model. These Exos were secreted by MSCs isolated from young mice. This report coincided with the findings presented here. However, in the present study, we did not further investigate whether the effects of Exos would decrease with senescence of secreting cells.

Next, we sought to identify the biological macromolecules that contributed to the varied effects between Aged- or Young- Exos. Therefore, miRNA array analysis was performed and more than 100 miRNAs were found to be differentially expressed. Specifically, in Aged-Exos, the content of 84 miRNAs were twice more than those in Young-Exos, and the content of 40 miRNAs was found to be less than the half of those in Young-Exos. The top ten miRNAs contained in Aged-Exos were then listed, and it was found that miR-128-3p was most upregulated.

Next, the potential downstream target genes of miR-128-3p were investigated. According to the database Targetscan, several candidate genes were reportedly associated with osteogenesis, such as BMP3, Smad9 and Smad5. With the administration of miR-128-3p inhibitor or mimics, BMP3 and smad9 results are not significant in the protein levels. In addition, Smad5 is the classical protein of osteogenic pathway. Among these genes, we took the most interest in Smad5. BMP groups were one of the three subfamilies of the transforming growth factor-β (TGF-β) family [55, 56]. The Smad1, 2, 3, 5, and 8 (or 9) are collectively referred to as receptor-regulated-Smads (R-Smads). Smad1/5/8, (maybe 9) are regulated through BMP receptor complexes [56–58]. Previous studies demonstrated that BMP2/4 transmitted signals via Smad1/5/8, and Smad5 was involved in the enhancement of cellular ALP activity induced by BMP6 [59, 60]. Furthermore, various recent studies have revealed a pivotal role of Smad5 in the osteogenesis of MSCs. Smad5 has been considered a target of several miRNAs and long noncoding RNA [61–64]. Given this observation, we sought to identify whether Smad5 was a target of miR-128-3p using the Luciferase reporter assay. As shown in Fig. 6a, luciferase activity was attenuated by the co-transfection of miR-128-3p mimics and Wt-Smad5, but not with the Mut-Smad5. When Wt-Smad5 was incubated with miR-128-3p inhibitor, there was an enhanced luciferase activity. In addition, according to Western Blot results, the expression levels of Smad5 protein in MSCs was markedly upregulated after administration of miR-128-3P inhibitor for 14 days. Based on these findings, it was demonstrated that miR-128-3P negatively modulates Smad5 by directly binding its 3′UTRs and possibly inhibits the osteogenesis process both in vitro and in vivo.

In vivo and in vitro experiments were conducted to verify whether decreased Smad5 contributed to the delayed osteogenic process caused by upregulating miR-128-3p in Aged-Exos. Overexpressed miR-128-3p was achieved by transfection of miR-128-3p mimics into young MSCs. Then it was found that the osteogenesis-related proteins such as Runx2, Collagen I, and ALP were remarkably downregulated. This finding indicated that miR-128-3p suppressed the osteogenic ability of MSCs in vitro. However, overexpressing Smad5 could reverse the inhibitory functions caused by miR-128-3p, which means miR-128-3p exerted its effects through Smad5. However, silencing Smad5 also abolished the positive results delivered by the miR-128-3p inhibitor.

The miR-128-3p antagomir was constructed to investigated the effects of miR-128-3p on fracture healing in vivo. After 2 weeks of local injections of the miR-128-3p antagomir around the surgery area, rats showed a remarkable increase in fracture healing based on micro-CT images. The expression of Smad5 underwent notable increasement in the callus tissue harvested from rats treated with miR-128-3p antagomir. Meanwhile, the immunohistochemical results of increased Smad5 in miR-128-3p antagomir group supported qRT-PCR as well. All these assays confirmed a direct interaction between miR-128-3P and Smad5, and indicated their essential roles in the recovery process of fractures in vivo.

Exosomes derived from MSCs have been reported by numerous studies to exert their biological functions by delivering specific miRNAs to the target cells. A recent study showed that over-expressed miRNA-181a in MSC-derived exosomes influenced the inflammatory response after myocardial ischemia–reperfusion injury [65]. Another study demonstrated that Exos derived from miR-30b-3p-overexpressing MSCs protect against acute lung injury induced by lipopolysaccharides [66]. Furthermore, miR-100-5p-abundant exosomes derived from fat pad MSCs was confirmed to protect articular cartilage via suppressing mTOR levels in osteoarthritis [67]. The miRNA array analysis presented in this report demonstrated that miR-128-3P was highly expressed in Aged-Exos compared to Young-Exos. In addition, miR-128-3P could be transferred to the target MSCs after incubation with Aged-Exo. Several previous studies showed that miR-128-3P played an inhibitory role in various cancer cases. For example, it was reported that miR-128-3p suppressed breast cancer progression by targeting LIM domain kinase 1(LIMK1) [68]. Furthermore, miR-128-3p was demonstrated to inhibit glioma cell proliferation and differentiation through the IRS-1/PI3K/AKT signaling pathway [69]. Exosome-transmitted miR-128-3p could also exert its anticancer effect by increasing the chemosensitivity of oxaliplatin-resistant colorectal cancer [70]. However, as far as we know, there have been no reports about the specific role of miR-128-3p in the MSCs osteogenic differentiation and bone fracture healing process. Likewise, Bing et al. [71] conducted bioinformatics and microarray analysis of miRNAs using fracture calluses from aged female mice and found several highly expressed miRNAs, including miR-128-3p. However, in this study, the effects of miR-128-3p on fracture healing was not investigated. In the present study, through a series of loss- and gain- function experiments, it was confirmed that upregulation of Smad5 in vitro or in vivo could abolish the adverse effects of miR-128-3p in the process of bone fracture healing. Smad5 was reported to take part in BMP-induced osteogenesis in various studies. Even without BMP2, Smad5 could still induce the expression of ALP and suppress myogenin-CTA activity after transfection into C2C12 myoblasts [72]. Type 1 collagen (Col I), alkaline phosphatase (ALP), and Runt related gene 2 (Runx2) are the necessary transcription factors for the osteogenic process and are responsible for the activation of OB differentiation marker genes. Runx2 is related to BMP2 signal transduction by interacting with Smad5, and coordination with BMP2-induced Smad5 is essential for Runx2 to activate osteogenesis [73, 74]. In our study, after the inhibition of miR-128-3p, the Smad5 expression was remarkably elevated and consequently, all these osteogenic marker genes were significantly upregulated, indicating that the osteogenesis process was enhanced.

## Conclusion

In this study, we demonstrated for the first time a novel phenomenon that Aged-Exos had diminished repair ability for bone fracture in rats. It was confirmed that miR-128-3p expression levels in MSCs, and their secreted Exos, augment as cell senescence arises. By suppressing Smad5 expression, the enriched levels of miR-128-3p in Aged-Exos attenuate the therapeutic effect and hold back the fracture healing process. These results not only supported the therapeutic effects of Young-Exos, but also demonstrated that Smad5 is one of the downstream target genes of exosomal miR-128-3p. There were other candidate genes that were not yet tested and verified in our study such as Smad9 or BMP3. These genes may also act as the target of miR-128-3p and may exert the synergistic effects with Smad5 on fracture healing. Thus, the antagomir for miR-128-3p may work as a novel and effective approach for bone fractures of aged patients.

## Supplementary information

**Additional file 1: Figure S1.** The Exos were absorbed by MSCs (scale: 20 μm).

**Additional file 2: Figure S2.** Wild-type Smad5 and mutant-type Smad5 luciferase reporter gene vector was constructed.

**Additional file 3: Figure S3.** The transfection efficiency of the mimics and the inhibitor was verified by qRT-PCR.

**Additional file 4.** miRNA Array Analysis of Young and Aged Exos.

## Data Availability

Materials described in the manuscript, including all relevant raw data, will be freely available to any scientist wishing to use them for non-commercial purposes, without breaching participant confidentiality.

## Abbreviations

MSCs: Mesenchymal stem cells
Exos: Exosomes
miRNAs: Micro-RNAs
NKIRAS2: NF-κB inhibitor interacting RAS-like 2
BMPs: Bone morphogenetic proteins
Smad5: Smad family member 5
SD rats: Sprague–Dawley rats
FBS: Fetal bovine serum
ALP staining: Alkaline Phosphatase staining
micro-CT: Microcomputed tomography
CV: Callus volume
BV: Bone volume
TV: Total volume
EDTA: Ethylenediaminetetraacetic acid
Col I: Collagen I
Runx2: Runt-related transcription factor 2
BCA: Bicinchoninic acid
PVDF: Poly-vinylidene fluoride
TBST: Tris-Buffered Saline Tween-20
GAPDH: Glyceraldehyde-3-phosphate dehydrogenase
NC-mimics: miR-128-3p-mimics-NC
NC-inhibitor: miR-128-3p-inhibitor-NC
NC-antagomir: miR-128-3p-antagomir-NC
DAB: 3,3-diaminobenzidine
ANOVA: Analysis of Variance
DLS: Dynamic light scattering
VEGF: Vascular endothelial growth factor
TGF-β: Transforming growth factor-β
