# Supplementary figures and images for: Exosomal miRNA-128-3p from mesenchymal stem cells of aged rats regulates osteogenesis and bone fracture healing by targeting Smad5
Source: J Nanobiotechnology. 2020 Mar 16;18:47. doi: 10.1186/s12951-020-00601-w (PMC7077029; doi:10.1186/s12951-020-00601-w)

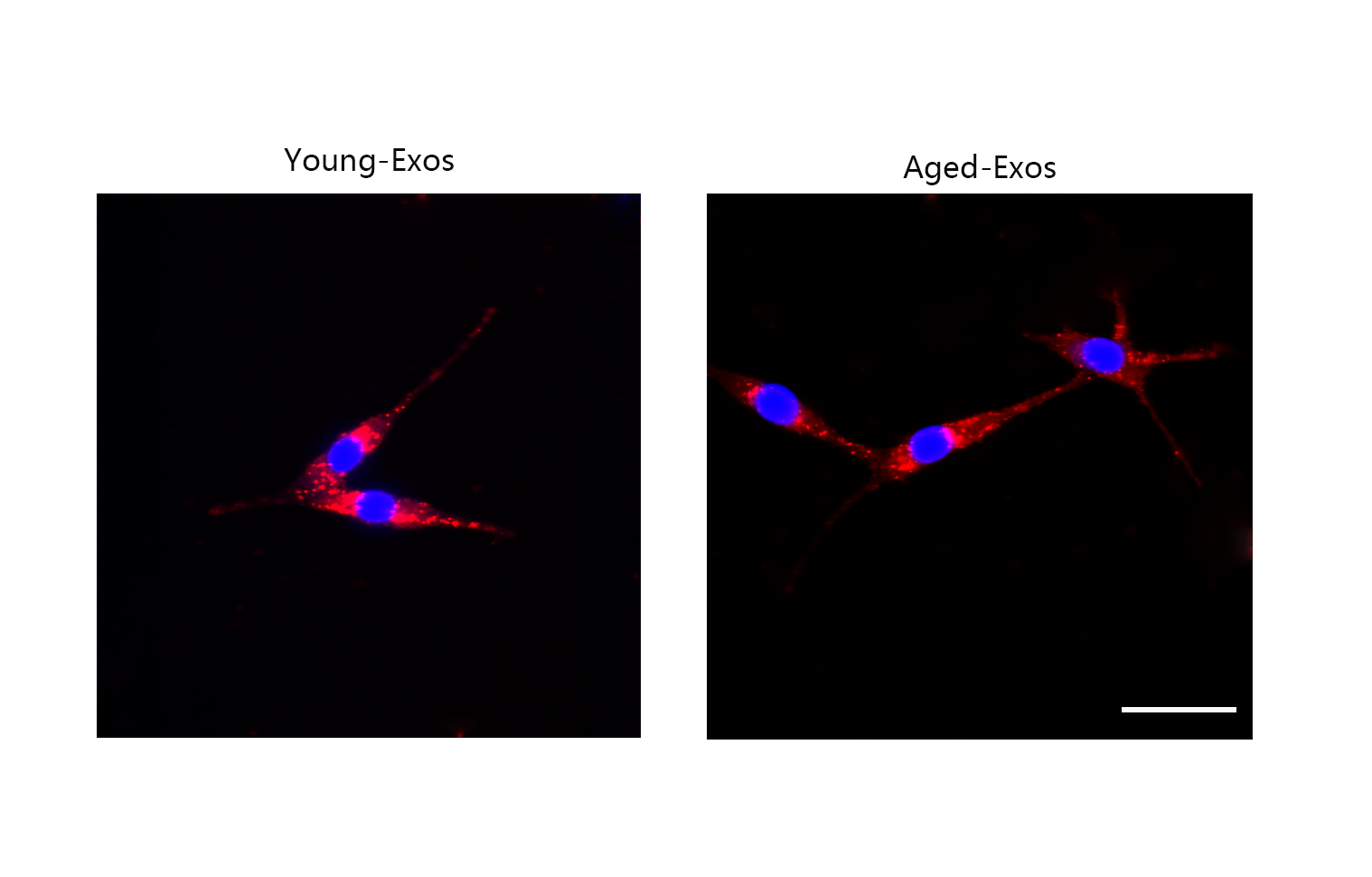

Supplement: Supplementary file 1 — Additional file 1: Figure S1. The Exos were absorbed by MSCs (scale: 20 μm). [file 12951_2020_601_MOESM1_ESM.tif]

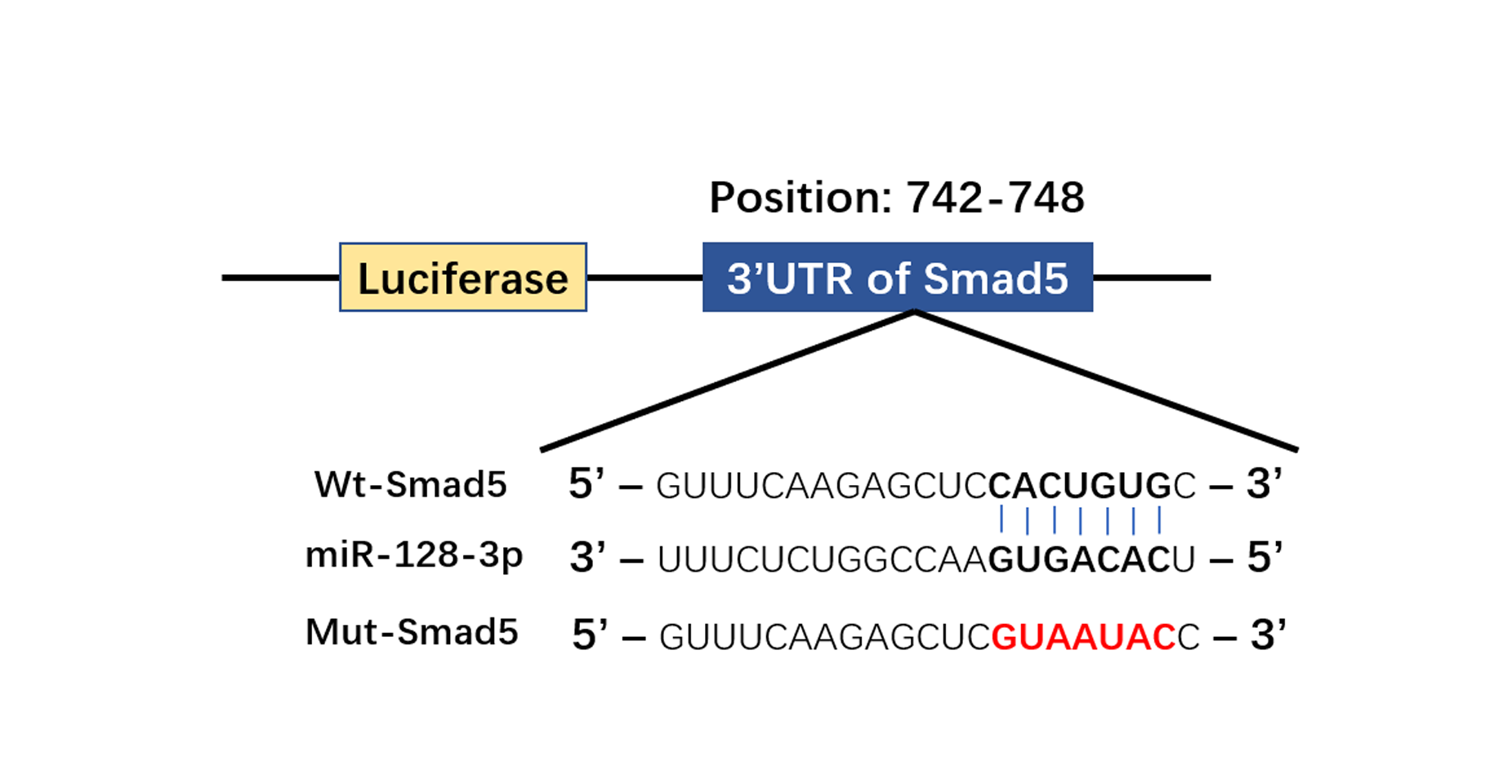

Supplement: Supplementary file 2 — Additional file 2: Figure S2. Wild-type Smad5 and mutant-type Smad5 luciferase reporter gene vector was constructed. [file 12951_2020_601_MOESM2_ESM.tif]

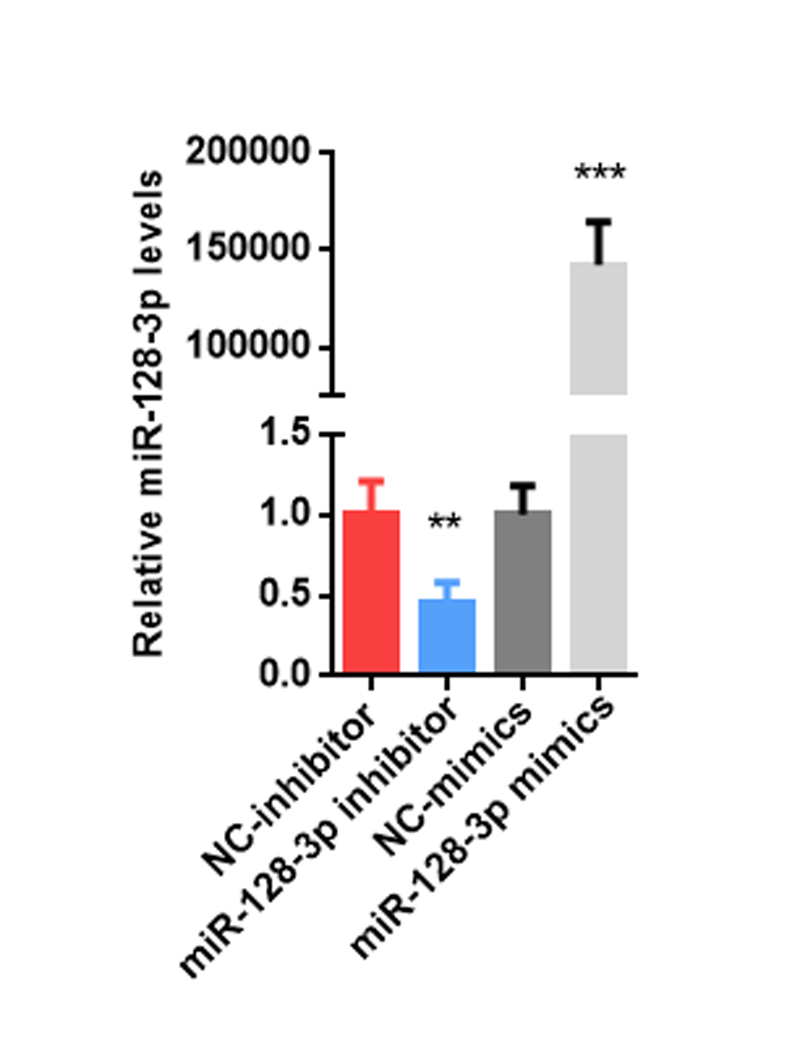

Supplement: Supplementary file 3 — Additional file 3: Figure S3. The transfection efficiency of the mimics and the inhibitor was verified by qRT-PCR. [file 12951_2020_601_MOESM3_ESM.tif]
